# Implementation of targeted cholera response activities, Cameroon

**DOI:** 10.2471/BLT.22.288885

**Published:** 2023-01-18

**Authors:** Jean Patrick Ouamba, Nicole Fouda Mbarga, Iza Ciglenecki, Ruwan Ratnayake, Dora Tchiasso, Flavio Finger, Nicolas Peyraud, Iscander Mounchili, Thierry Boyom, Cyrille Yonta, Liliane Nwatchok, Moustapha Mouhamadou, Christine Ekedi, Johne Marcel, Francisco Luquero, Faustin Ekah, Adidja Amani, Modeste Tamakloe, Yap Boum, Linda Esso

**Affiliations:** a Médecins Sans Frontières, Yaounde, Cameroon.; b World Health Organization, PO Box 155, Yaounde, Cameroon.; c Médecins Sans Frontières, Geneva, Switzerland.; d Epicentre, Paris, France.; f Kribi District Hospital, Regional Delegation of the South, Kribi, Cameroon.; g Médecins Sans Frontières, Dakar, Senegal.; h United Nations International Children's Emergency Fund, Yaounde, Cameroon.; i Sub-direction of Vaccination, Ministry of Public Health, Yaounde, Cameroon.; j Epicentre, Yaounde, Cameroon.; k Department for the Control of Disease, Epidemics and Pandemics, Ministry of Public Health, Yaounde, Cameroon.

## Abstract

**Objective:**

To describe the implementation of case-area targeted interventions to reduce cholera transmission using a rapid, localized response in Kribi district, Cameroon.

**Methods:**

We used a cross-sectional design to study the implementation of case-area targeted interventions. We initiated interventions after rapid diagnostic test confirmation of a case of cholera. We targeted households within a 100–250 metre perimeter around the index case (spatial targeting). The interventions package included: health promotion, oral cholera vaccination, antibiotic chemoprophylaxis for nonimmunized direct contacts, point-of-use water treatment and active case-finding.

**Findings:**

We implemented eight targeted intervention packages in four health areas of Kribi between 17 September 2020 and 16 October 2020. We visited 1533 households (range: 7–544 per case-area) hosting 5877 individuals (range: 7–1687 per case-area). The average time from detection of the index case to implementation of interventions was 3.4 days (range: 1–7). Oral cholera vaccination increased overall immunization coverage in Kribi from 49.2% (2771/5621 people) to 79.3% (4456/5621 people). Interventions also led to the detection and prompt management of eight suspected cases of cholera, five of whom had severe dehydration. Stool culture was positive for *Vibrio cholerae* O1 in four cases. The average time from onset of symptoms to admission of a person with cholera to a health facility was 1.2 days.

**Conclusion:**

Despite challenges, we successfully implemented targeted interventions at the tail-end of a cholera epidemic, after which no further cases were reported in Kribi up until week 49 of 2021. The effectiveness of case-area targeted interventions in stopping or reducing cholera transmission needs further investigation.

## Résumé

**Objectif:**

Décrire la mise en œuvre d'interventions ciblées, dans les zones de survenue des cas, dont le but consiste à limiter la transmission du choléra en réagissant de manière rapide et localisée dans le district de Kribi, au Cameroun.

**Méthodes:**

Nous avons utilisé un modèle transversal afin d'étudier la mise en œuvre d'interventions ciblées dans les zones de survenue des cas. Nous avons lancé des actions après confirmation d'un cas de choléra par test de diagnostic rapide. Nous nous sommes focalisés sur les ménages situés dans un périmètre de 100–250 mètres autour du cas indicateur (ciblage spatial). Le module d'intervention prévoyait: la promotion de la santé, la vaccination orale contre le choléra, une chimioprophylaxie antibiotique pour les contacts directs non immunisés, le traitement de l'eau au point d'utilisation et la détection active de cas.

**Résultats:**

Nous avons déployé huit modules d'intervention ciblée dans quatre secteurs de santé de Kribi entre le 17 septembre 2020 et le 16 octobre 2020. Nous nous sommes rendus auprès de 1533 ménages (plage comprise entre 7 et 544 par zone de survenue de cas) représentant 5877 individus (plage comprise entre 7 et 1687 par zone de survenue de cas). La durée moyenne entre la détection du cas indicateur et l'organisation d'interventions était de 3,4 jours (plage comprise entre 1 et 7). La vaccination orale contre le choléra a amélioré la couverture vaccinale globale à Kribi, celle-ci passant de 49,2% (2771/5621 personnes) à 79,3% (4456/5621 personnes). Les interventions ont également entraîné une détection et une prise en charge rapide de huit cas présumés de choléra, dont cinq souffraient de déshydratation sévère. La culture de selles s'est révélée positive à *Vibrio cholerae* O1 dans quatre cas. La durée moyenne entre l'apparition des symptômes et l'admission d'une personne atteinte du choléra dans un établissement de santé s'élevait à 1,2 jour.

**Conclusion:**

Malgré les défis, nous avons réussi à mettre en œuvre des interventions ciblées à la fin d'une épidémie de choléra. Par la suite, aucun autre cas n'a été signalé à Kribi jusqu'à la 49^e^ semaine de 2021. L'efficacité des interventions ciblées dans les zones de survenue des cas pour diminuer ou stopper la transmission du choléra doit être examinée plus en détail.

## Resumen

**Objetivo:**

Describir la implementación de intervenciones selectivas en el área de casos para reducir la transmisión del cólera utilizando una respuesta rápida y localizada en el distrito de Kribi, Camerún.

**Métodos:**

Se utilizó un diseño transversal para estudiar la implementación de intervenciones selectivas en el área de casos. Se iniciaron las intervenciones tras la confirmación de un caso de cólera mediante una prueba de diagnóstico rápido. Las intervenciones se centraron en los hogares situados en un perímetro de entre 100 y 250 metros alrededor del caso inicial (selección espacial). El paquete de intervenciones incluía: promoción de la salud, vacunación oral contra el cólera, quimioprofilaxis antibiótica para los contactos directos no inmunizados, tratamiento del agua en los puntos de consumo y búsqueda activa de casos.

**Resultados:**

Entre el 17 de septiembre y el 16 de octubre de 2020, se implementaron ocho paquetes de intervención selectiva en cuatro áreas de salud de Kribi. Se visitaron 1533 hogares (rango: de 7 a 544 por área de casos) que acogían a 5877 personas (rango: de 7 a 1687 por área de casos). El tiempo medio transcurrido desde la detección del caso inicial hasta la implementación de las intervenciones fue de 3,4 días (rango: de 1 a 7). La vacunación por vía oral contra el cólera aumentó la cobertura global de inmunización en Kribi del 49,2 % (2771/5621 personas) al 79,3 % (4456/5621 personas). Las intervenciones también permitieron detectar y tratar con rapidez ocho casos sospechosos de cólera, cinco de los que presentaban deshidratación grave. El cultivo de heces dio positivo para *Vibrio* *cholerae* O1 en cuatro casos. El tiempo medio transcurrido desde el inicio de los síntomas hasta el ingreso de una persona con cólera en un centro sanitario fue de 1,2 días.

**Conclusión:**

A pesar de los desafíos, se implementaron con éxito las intervenciones selectivas al final de una epidemia de cólera, después de lo cual no se registraron más casos en Kribi hasta la semana 49 de 2021. Es necesario seguir investigando la eficacia de las intervenciones selectivas en el área de casos para detener o reducir la transmisión del cólera.

## ملخص

**الغرض:**

وصف تنفيذ التدخلات المستهدفة في منطقة الحالة للحد من انتشار الكوليرا باستخدام استجابة محلية سريعة في منطقة كريبي بالكاميرون.

**الطريقة:**

قمنا باستخدام تصميم مقطعي لدراسة تنفيذ التدخلات المستهدفة لمنطقة الحالة. بدأنا التدخلات بعد تأكيد الاختبار التشخيصي السريع لإحدى حالات الكوليرا. استهدفنا الأسر في محيط 100 إلى 250 مترًا حول الحالة المرجعية (الاستهداف المكاني). تضمنت حزمة التدخلات: تعزيز الصحة، والتطعيم الفموي ضد الكوليرا، والوقاية الكيميائية بالمضادات الحيوية من أجل الأطراف المتصلة غير المحصنة، ومعالجة المياه في نقطة الاستخدام، والاكتشاف النشط للحالات.

**النتائج:**

قمنا بتنفيذ ثماني حزم من التدخلات المستهدفة في أربع مناطق صحية في كريبي بين 17 سبتمبر/أيلول 2020، و16 أكتوبر/تشرين أول 2020. قمنا بزيارة 1533 أسرةً (المدى: 7 إلى 544 لكل منطقة حالة) تستضيف 5877 فردًا (المدى: 7 إلى 1687 لكل منطقة حالة). كان متوسط الوقت من اكتشاف الحالة المرجعية إلى تنفيذ التدخلات 3.4 يومًا (المدى: 1 إلى 7). أدى التطعيم الفموي ضد الكوليرا إلى زيادة تغطية التحصين الشاملة في كريبي من %49.2 (2771/5621 شخصًا) إلى %79.3 (4456/5621 شخصًا). كما أدت التدخلات إلى الكشف عن ثماني حالات مشتبه بإصابتها بالكوليرا ومعالجتها بسرعة، خمسة منها تعاني من الجفاف الشديد. كانت مزرعة البراز إيجابية بالنسبة لبكتيريا الكوليرا O1 في أربع حالات. كان متوسط الوقت منذ ظهور الأعراض حتى دخول شخص مصاب بالكوليرا إلى مرفق صحي 1.2 يومًا.

**الاستنتاج:**

على الرغم من التحديات التي نواجهها، فقد نجحنا في تنفيذ التدخلات المستهدفة في نهاية وباء الكوليرا، والتي بعدها لم يتم الإبلاغ عن حالات أخرى في كريبي حتى الأسبوع 49 من عام 2021. إن فعالية التدخلات المستهدفة لمنطقة الحالة في إيقاف انتشار الكوليرا، أو الحد منها، بحاجة لمزيد من التحقيقات.

## 摘要

**目的:**

描述在喀麦隆克里比区通过快速的局部应对措施，实施针对病例区的干预措施，以减少霍乱传播的情况。

**方法:**

我们采用横断面设计来研究病例区针对性干预措施的实施情况。我们在快速诊断检测确认一例霍乱病例后开始采取干预措施。我们以指标病例周围 100-250 米范围内的家庭为目标（空间定位）。一揽子干预措施包括：健康宣传、口服霍乱疫苗、非免疫直接接触者的抗生素化学预防、使用点水处理和主动筛检病例。

**结果:**

2020 年 9 月 17 日至 10 月 16 日期间，我们在克里比区的四个卫生领域实施了八项有针对性的干预计划。我们走访了 1533 户家庭（范围：每个病例区 7-544 户），共接待 5877 人（范围：每个病例区 7-1687 人）。从发现指示病例到实施干预措施的平均时间为 3.4 天（范围：1-7 天）。口服霍乱疫苗使克里比区的总体免疫覆盖率从 49.2%（2771/5621 人）提高到 79.3%（4456/5621 人）。干预措施还发现并及时处理了 8 例疑似霍乱病例，其中 5 例出现严重脱水。4 例患者粪便培养 O1 *群霍乱弧菌*呈阳性。霍乱患者从出现症状到被送往卫生机构的平均时间为 1.2 天。

**结论:**

尽管面临挑战，我们还是成功地在霍乱流行末期实施了有针对性的干预措施，此后直到 2021 年第 49 周，克里比区再无新增病例报告。针对病例区的干预措施在阻止或减少霍乱传播方面的有效性需要进一步调查。

## Резюме

**Цель:**

Описать процесс проведения целевых мероприятий по снижению заболеваемости холерой в районе Криби, Камерун, с использованием экстренных локализованных ответных мер.

**Методы:**

Для исследования проведения целевых мероприятий на конкретных территориях использовался перекрестный метод. Меры были приняты после подтверждения случая заболевания холерой по результатам экспресс-диагностики. Объектом исследования выступали домохозяйства в радиусе 100–250 метров вокруг источника заболевания (пространственный таргетинг). В комплекс мероприятий входили: мероприятия по укреплению здоровья, пероральная вакцинация против холеры, химиопрофилактика антибиотиками для прямых контактов неиммунизированных лиц, обработка воды в местах потребления и активное выявление случаев заболевания.

**Результаты:**

В период с 17 сентября по 16 октября 2020 года было проведено восемь целевых мероприятий в четырех медицинских районах Криби. Были посещены 1533 домохозяйства (диапазон: 7–544 в каждом районе), в которых проживало 5877 человек (диапазон: 7–1687 в каждом районе). Среднее время от выявления источника заболевания до проведения мероприятий составило 3,4 дня (диапазон: 1–7). При пероральной вакцинации против холеры общий охват иммунизацией в Криби увеличился с 49,2% (2771/5621 человек) до 79,3% (4456/5621 человек). Принятые меры также позволили выявить и своевременно провести лечение восьми предполагаемых случаев холеры, в пяти из которых наблюдалось сильное обезвоживание. В 4 случаях посев кала на штаммы *Vibrio cholerae* O1 был положительным. Среднее время от появления симптомов до поступления больного холерой в медицинское учреждение составило 1,2 дня.

**Вывод:**

Несмотря на трудности, в конце эпидемии холеры были успешно проведены целевые мероприятия, после которых вплоть до 49-й недели 2021 года в Криби больше не было зарегистрировано ни одного случая заболевания. Эффективность целевых мероприятий по прекращению или сокращению передачи холеры в исследуемом районе требует дальнейшего изучения.

## Introduction

Cholera is an infectious disease which causes acute watery diarrhoea as a result of ingestion of water or food contaminated with *Vibrio cholerae*.^1^^–^^3^ Cholera remains a global health challenge with the greatest burden in Africa.^2^ For severe cases and without timely treatment, cholera quickly causes severe dehydration and death.^4^ Within a short period following the emergence of a cholera case in a community, there is an increased risk of cholera among household contacts,^5^^–^^7^ neighbours and people living within a range of 100–250 metres of the infected person.^8^^,^^9^

Case-area targeted interventions refer to interventions in neighbourhoods of people with cholera, which may include health promotion, oral cholera vaccination, point-of-use water treatment and antibiotic chemoprophylaxis.^10^ There is scarce evidence of the effectiveness of targeted interventions in the control of cholera epidemics. A modelling study of targeted interventions (oral cholera vaccination, antibiotics and point-of-use water treatment) observed a reduction in cholera transmission when used as a complement to other interventions.^11^ A relationship was observed between the speed of implementing targeted interventions (without oral cholera vaccination) and a reduction in the incidence of suspected cholera cases and of outbreak duration in Haiti from 2015–2017.^10^ In Africa, the feasibility of targeted interventions without oral cholera vaccination was described in the Democratic Republic of Congo, in South Sudan^11^^–^^14^ and in Cameroon.^15^ Targeted spatiotemporal interventions implemented in the neighbourhood of cholera patients in 2004 in Douala, Cameroon, were followed by a reduction in the incidence of cholera among contacts of cases.^15^ Case-area targeted interventions have shown promising results regarding cholera outbreak control.^16^

In May 2020 (week 18), a cholera epidemic started in the Kribi health district in the South Region of Cameroon.^17^ By August 2020 (week 31), a full cholera response was in place. The interventions included: free-of-charge case management; strengthening surveillance; risk communication and community engagement; water, sanitation and hygiene activities (water quality tests, disinfection of households); and a reactive, mass oral cholera vaccination campaign in one health area (week 32) with extension to four health areas (week 36).^18^ Despite these interventions, clusters of cases were still reported, especially in slums and at the central prison where oral cholera vaccination coverage was suboptimal.^19^ These outbreaks prompted implementation of a rapid, localized response. Given the lack of operational research on case-area targeted interventions, we aimed to describe how targeted interventions with oral cholera vaccination were systematically implemented as complementary interventions to halt the spread of the cholera epidemic in Kribi.

## Methods

### Setting and population

Kribi is a cosmopolitan, seaside town located in the South Region of Cameroon, a major tourist centre. Kribi health district consists of 11 health areas with a population of 154 370 inhabitants.^18^ The target population for the study included all people living within a specified radius of an index cholera case, with the radius depending on the population density. For oral cholera vaccination, all persons aged at least 1 year old were eligible. We implemented case-area targeted interventions in Kribi over a period of 1 month from 16 September 2020 (week 38) to 17 October 2020 (week 42).

### Strategy

We used a multistage implementation strategy. Stage 1 consisted of preparedness for case-area targeted interventions which was triggered after a report of at least one case of acute watery diarrhoea who tested positive for cholera by enriched rapid diagnostic test.^20^ Enrichment of stool specimen with alkaline peptone water increases specificity by up to 97%^20^ and reduced the likelihood of obtaining false-positive results.^21^ We tested stool samples using rapid diagnostic test kits Crystal® VC (Cholkit, Arkray Health Care Pvt. Ltd, Mumbai, India) or SD Bioline Cholera Ag (Standard Diagnostics Inc., Gyeonggi-do, Republic of Korea). For confirmation, we sent Cary-Blair swab samples to the national public health laboratory in the capital city, Yaounde, for culture as part of an ongoing study on the performance of cholera rapid diagnostic tests. 

Stage 2 was the launch of case-area targeted interventions within 7 days after identification of a cholera case confirmed by enriched rapid diagnostic testing. We informed community leaders before we started any activities in the community and asked them to approve all activities to be undertaken. The main elements of the approach included rapid deployment of a team who visited the household of the index case and discussed the interventions with community leaders. Using a global positioning system (GPS)-enabled tablet computer, the team identified the GPS coordinates of households hosting cholera cases and determined the radius required for the interventions. Field staff also enumerated households and inhabitants before interventions started. Active case-finding involved a search for other people with symptoms of cholera in the same household and in households within the target-area radius. When other cases were detected, the patient was offered oral rehydration solution and referred to the cholera treatment unit in Kribi district hospital if the person was moderately or severely dehydrated.

Stage 3 involved administering interventions in the household of the index case and other households within the target area. In the index household, the interventions included health promotion, water, sanitation and hygiene measures, vaccination and prophylactic antibiotic therapy. The health promotion component comprised education on basic hygiene measures and actions to be taken in case of diarrhoea. The water, sanitation and hygiene intervention included an assessment of the community water source and the availability of toilets; distribution of a minimum number of two packs of chlorine tablets for water purification and five soap bars (350 g) for a period of 1 month; and distribution of water storage containers, where possible. We administered oral cholera vaccination with a single dose of Euvichol® (uBiologics, Seoul, Republic of Korea) to all inhabitants of the household aged at least 1 year old who had not been vaccinated during the mass campaign. Prophylactic antibiotic therapy was designed to reduce the household members’ risk of developing severe clinical forms of cholera and to rapidly reduce onward transmission. We offered an oral antibiotic (azithromycin or doxycycline) to all people in the household aged at least 1 year old and who had no history of administration of oral cholera vaccination. We used a door-to-door strategy to deliver interventions to households within the target radius. We offered the same package of interventions (except for antibiotic prophylaxis) to households and residents residing within a radius of 100 to 250 m around the household of the index case, depending on the population density.

### Teams and resources

Each case-area targeted interventions team comprised an epidemiologist, an investigator, a water, sanitation and hygiene specialist, a health promotion supervisor and community relay agents who were all national staff from *Médecins Sans Frontières* and the Cameroon Ministry of Public Health. Depending on the size of the targeted population, the number of community health workers varied from six to 10 per targeted intervention (approximately one relay agent for 100 people). The exact number of people in the team ranged from 10 to 14. The list of materials used for implementation of interventions in the target areas can be found in the online repository.^22^

### Data collection and analysis

We used a cross-sectional study design to describe the case-area targeted interventions, their coverage and potential effects. Data sources included: (i) the patient list for those admitted to health-care facilities; (ii) the household contact list held by the intervention team; and (iii) the data collected within the target area by the intervention team, including coverage of interventions. Intervention coverage was estimated as the proportion of people and households who received the interventions within a given target-area radius. The main variables and outcomes were: promptness of the response; the proportion of people or households who received the interventions (health education, vaccination, antibiotic chemoprophylaxis and point-of-use water treatment); and the incidence of cholera in the target areas over time. A trained epidemiologist supervised the data collection. Data were entered into tablets using Kobo software (Kobo Inc., Toronto, Canada) to reduce the potential for errors. 

For the analysis, we expressed continuous variables as means and simple ranges and summarized categorical variables (coverage) as counts and percentages. We constructed an epidemic curve to visualize the incidence of cases and timeline of interventions. We compiled the direct and indirect costs of the intervention (human resources, equipment and vaccines). We used Microsoft Excel (Microsoft Corp., Redmond, United States of America) for data analysis.

### Ethical considerations

Case-area targeted interventions were validated as complementary interventions to cholera control by the Department for the Control of Disease, Epidemics and Pandemics of the Cameroon Ministry of Public Health. Administrative clearance was obtained from local authorities. All families who took part in this intervention gave verbal consent. Only aggregated data collected for programmatic purposes were used for this analysis and no individual-level patient data were used.

## Results

Through active case-finding in the Kribi health area, nine cases of cholera were identified by rapid diagnostic testing (including one death in the community). These cases triggered implementation of eight case-area targeted interventions in seven neighbourhoods of four health areas of the Kribi district over 1 month (Table 1). Contacts of one index case could not be traced, therefore interventions could not be implemented for that person. Most interventions were in urban settings (five out of eight) and one intervention took place at the central prison. The average time between case detection and complete implementation of the interventions was 3.4 days (range: 1.0–7.0). On average, an area covered in a targeted intervention had a radius of about 150 m (range: 100–250), covering from seven to 544 households and seven to 1687 individuals (Table 1), depending on the population density. The average number of people per household was 3.8 (range: 1.0–12.6).

**Table 1:** Demographic characteristics of sites of case-area targeted interventions for cholera, Kribi, Cameroon, 2020

Community or village	Health area	Setting	Radius of target area, metres	No. of households enumerated	No. of people enumerated	Mean no. of people per household or block	Duration of interventions, days	Time from case detection to start of interventions, days
Afan Mabe	Kribi	Urban	100	288	1553	5.4	3	6
Central prison	Kribi	Urban	250	30 (+11 blocks)^a^	377	12.6	1	1
Damakale	Elog Batindi	Rural	250	7	7	1.0	1	1
Mbeka'a Paris	Grand Batanga	Rural	250	120	388	3.2	1	2
Mokolo	Kribi	Urban	250	315	1096	3.5	2	7
Petit Paris	Kribi	Urban	100	544	1687	3.1	1	6
Wamié	Kribi	Urban	100	113	386	3.4	1	3
Village 7	Hevecam	Rural	100	116	383	3.3	2	1

^a^ The Central prison community included 30 households in the prison surroundings, which were covered by the target radius of 150 m, plus 11 prison blocks.

### Clinical characteristics 

The mean age of the eight people with cholera was 25 years (range: 6 weeks to 66 years) and five of them were male. One in two individuals had a positive stool culture for *V. cholerae.* The strain most commonly identified through culture was *V. cholerae* serotype O1 (four out of eight people). Among the index cases, resistance for doxycycline was identified in one out of four people tested and no resistance for azithromycin was reported. Five out of eight people had severe dehydration (Table 2).

**Table 2:** Clinical characteristics of cholera cases identified through case-area targeted interventions, Kribi, Cameroon, 2020

Community or village	Age	Gender	Symptoms	Rapid diagnostic test results	Culture results	Level of dehydration^a^	Antibiogram result
Afan Mabe	66 years	Female	Diarrhoea, vomiting	Positive cholera O1	Positive *Vibrio cholerae* O1	Severe	Sensitive: gentamicin, ciprofloxacin, chloramphenicol. Resistant: amoxicillin, erythromycin, amoxicillin + clavulanic acid, nalidixic acid, colistin, tetracycline doxycycline, cephalothin, polymyxin B
Central prison	31 years	Male	Diarrhoea, vomiting	Positive cholera O1	Negative	None	NA
Damakale	38 years	Male	Diarrhoea, vomiting	Positive cholera O1	Positive *Vibrio cholerae* non-O1, non-O139	Mild	Sensitive: gentamicin, ciprofloxacin, doxycycline, chloramphenicol, erythromycin, cotrimoxazole, azithromycin, ofloxacin. Resistant: amoxicillin, amoxicillin + clavulanic acid, nalidixic acid, streptomycin.
Mokolo	19 years	Male	Diarrhoea, vomiting	Positive cholera O1	Positive *Vibrio cholerae* O1	Severe	Sensitive: gentamicin, ciprofloxacin streptomycin, ofloxacin, azithromycin, cefotaxime, doxycycline, erythromycin, chloramphenicol, cotrimoxazole. Resistant: amoxicillin, amoxicillin + clavulanic acid, nalidixic acid.
Mbeka’a Paris	2 years	Male	Diarrhoea, vomiting	Positive cholera O1	NA	Severe	NA
Petit Paris	6 weeks	Male	Diarrhoea, vomiting	Positive cholera O1	Negative	Severe	NA
Village 7	25 years	Female	Diarrhoea, vomiting, severe dehydration (death)	Positive cholera O1	NA	Severe	NA
Wamié	26 years	Female	Diarrhoea, vomiting	Positive cholera O1	Positive *Vibrio cholerae* O1	None	Sensitive: gentamicin, ciprofloxacin, streptomycin, doxycycline chloramphenicol, cotrimoxazole. Intermediate: erythromycin, azithromycin. Resistant: amoxicillin, amoxicillin + clavulanic acid, cefotaxime, nalidixic acid, erythromycin, polymyxin B, colistin.

NA: data not available.

^a^ Level of dehydration categories were those of the World Health Organization.^23^

Overall, 1685 people were vaccinated in five of eight communities where targeted interventions were implemented. The highest proportion of people were immunized at the central prison (314/377 people, 83.3%), followed by Mokolo community (509/1054 people, 46.4%). Vaccines were not administered to the last three targeted interventions in Damakale, Mbeka’a Paris and Village 7 because of vaccine shortages. Across all sites, 86.2% of households (1322/1533 households) were reached by health promotion, ranging from 76.1% (86/113 households) in Wamié to 100.0% (30/30 households) in the central prison. All households reached received chlorine tablets and soap bars. A total of 18 824 packs of chlorine tablets was distributed, especially in the communities of Mokolo (4400 packs) and Petit Paris (6992 packs). The highest number of people receiving antibiotics as prophylaxis were at the central prison (73 people), Damakale (seven people) and Village 7 (seven people; Table 3).

**Table 3:** Summary of components of the case-area targeted interventions for cholera in the Kribi health district, Cameroon, 2020

Community or village	No. of households enumerated	No. (%) of households reached for health promotion	No. of people enumerated	No of people given oral cholera vaccination	No. of chlorine tablet packs distributed to households^a^	No. of soap bars distributed to households^a^	No. of people started on chemoprophylaxis in households of index case
Afan Mabe	288	280 (97)	1553	564	2800	1680	2
Central prison	30 (+11 blocks)^b^	30 (100)	377	313	480	180	73
Damakale	7	7 (100)	7	0	112	42	1
Mbeka’a Paris	120	99 (82)	388	0	1584	594	7
Mokolo	315	275 (87)	1096	509	4400	1650	1
Petit Paris	544	437 (80)	1687	204	6992	2622	0
Village 7	116	108 (93)	383	0	1080	648	7
Wamié	113	86 (76)	386	95	1376	516	1

^a^ All households within the radius of the target areas received chorine tablets and soap bars.

^b^ The Central prison community included 30 households in the prison surroundings, which were covered by the target radius of 150 m, plus 11 prison blocks.

### Response times

Across all targeted interventions, the average time elapsed from the onset of symptoms to admission of a person with suspected cholera was 1.0 day (range: 1.0–2.0). All suspected cholera patients admitted to a health facility had their rapid diagnostic test results within 1 hour. The average time between the rapid diagnostic test result and the launch of interventions was 3.4 days (range: 0.5 to 6.0 days). On average it took 1.5 days (range: 1.0–3.0) for all the components of the interventions to be completed in a target area.

### Vaccination coverage

Oral cholera vaccination was possible in five of the eight targeted interventions. The percentage of people newly vaccinated in the target areas ranged from 12.6% (204/1613 people) in Petit Paris to 83.0% (313/377 people) at the central prison (Table 3). Targeted interventions with oral cholera vaccination boosted average immunization coverage in areas at risk from 49.3% (2771/5621 people) to 79.3% (4456/5621 people). The highest final vaccination coverages in the Kribi health area were achieved in Mokolo (1043/1054 people; 98.9%) and the central prison (362/377 people, 96.0%; Table 4).

**Table 4:** Vaccination coverage in the target areas before and after case-area targeted interventions with one dose of oral cholera vaccination, Kribi, Cameroon, 2020

Community or village	No. of people enumerated	No. of people eligible for vaccination^a^	No. (%) of people previously vaccinated	No. (%) of people vaccinated during targeted interventions	Total no. (%) of people vaccinated
Afan Mabe	1553	1463	804 (54.9)	564 (38.5)	1368 (93.5)
Central prison	377	377	49 (12.9)	313 (83.0)	362 (96.0)
Damakale	7	7	0 (0.0)	0 (0.0)	0 (0.0)
Mbeka'a Paris	388	367	167 (45.5)	0 (0.0)	167 (45.5)
Mokolo	1096	1054	534 (50.6)	509 (48.2)	1043 (98.9)
Petit Paris	1687	1613	838 (51.9)	204 (12.6)	1042 (64.6)
Village 7	383	370	251 (67.8)	0 (0.0)	251 (67.8)
Wamié	386	370	128 (34.5)	95 (25.6)	223 (60.2)
**Total**	5877	5621	2771 (49.2)	1685 (30.0)	4456 (79.2)

^a^ All residents older than 1 year old in the risk areas were eligible for cholera vaccination.

### Epidemic curve

**Fig. 1:**
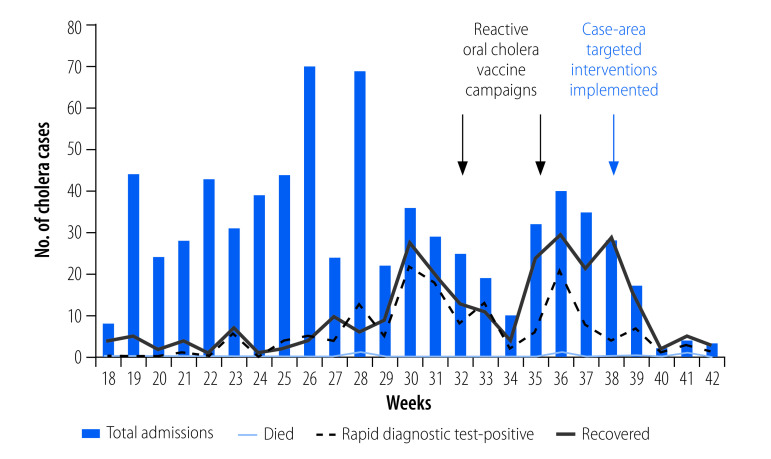
Epidemic curve of the incidence of cases and timeline of case-area targeted interventions for cholera, Kribi, Cameroon, 2020

Note: The figure shows the number of new cases of cholera identified and outcomes of patients from week 18 at the start of the cholera epidemic in to week 39 when we implemented case-area targeted interventions in eight target areas at the tail-end of the epidemic.

As shown in Fig. 1, implementation of targeted interventions during week 38 was followed by a decrease in the number of cases and a flattening of the curve at week 42. Interventions were stopped when no further rapid diagnostic test-confirmed cases were reported.

## Discussion

We found that it was feasible to implement case-area targeted interventions in a timely way at the tail-end of a cholera epidemic in Kribi district. The interventions were effective at boosting vaccination coverage in the target populations at risk in Kribi, delivering water, sanitation and hygiene interventions to people living in the neighbourhood and providing chemoprophylaxis to people most at risk. Targeted interventions also provided timely opportunities for active case-finding in the community and referrals for management. Although we could not establish the effect of targeted interventions on cholera incidence, we believe that the rapid implementation of targeted interventions contributed to the rapid decrease in transmission and containment of the outbreak. After the end of the study, no further cases were reported in Kribi up until week 49 of 2021.

Despite its originality, implementation of case-area targeted interventions come with several field challenges, including increased cost and promptness of actions which require robust coordination and collaboration. In addition to the limited supply of oral cholera vaccine and some people’s hesitancy to be vaccinated, the availability of the resources necessary for the interventions was a major limitation. In Kribi, targeted interventions were launched without availability of water, sanitation and hygiene items such as chlorine tablets; these were later made available by the United Nations Children’s Fund (UNICEF). Additionally, surveillance may have not detected some cases. Targeted interventions were clustered around cases and conducted in a relatively small area with adequate personnel, which may not reflect many settings where cholera outbreaks happen. Lastly, with the ongoing pandemic of coronavirus disease-2019 (COVID-19), there was less interest in cholera response activities among partner organizations. Nonetheless, the collaborative governance of the Cameroon health ministry led to mobilization of stakeholders and successful implementation of the interventions in Kribi.

In previous implementations, an association was observed between the promptness of initiating case-area targeted interventions and length of cholera outbreaks. Prompt targeted interventions were effective in shortening cholera epidemics in Haiti.^10^ In Kribi, apart from the first interventions performed on the seventh day after identification of a cholera case, all other targeted interventions were started within a period of less than 7 days. We benefited from an ongoing study on the performance of rapid diagnostic tests, which allowed for easy access to enriched rapid diagnostic tests, thus enabling rapid case detection and confirmation by culture. However, promptness of case presentation, outbreak detection, investigation and response are challenging during cholera outbreaks. In a meta-analysis of cholera outbreaks, the median delay between detection and response was 10 days (interquartile range: 7–18).^24^ Although there has been success in Haiti and Yemen,^10^^,^^25^ interventions teams can rapidly become overwhelmed and resources become depleted as small outbreaks progress to large outbreaks.^10^^,^^12^ For these reasons, targeted interventions appear to be less demanding when implemented at the end of an outbreak^10^^,^^12^ (as seen here in Kribi and in Juba in South Sudan^13^) but also promptly before the outbreak gets too large.

The choice of the radius for targeted interventions determines the efficacy of the interventions. A modelling study of 4352 reported cases in Chad over 232 days found that cholera cases were reduced by 81% and the length of cholera epidemics reduced by 63% when interventions were implemented with oral cholera vaccination and within a 100 m radius of index cases.^10^ In our settings, the choice of the radius for targeted interventions was based on the setting (rural or urban) and the density of households. The majority of interventions (five out of eight) were performed in the urban area of Kribi, which made implementation easier. In crowded areas such as the central prison, everyone in the vicinity of cases were included in the intervention due to concerns about equity, raising the radius of intervention to 250 m. In less crowded areas, the radius was also increased to 250 m maximum. Damakale had fewer people included in the interventions because it is a village with scattered households, with an average of one household within a radius of 100–250 m, even up to 1 km from the index case.

Several authors have described the added value of including oral cholera vaccination in case-area targeted interventions.^11^^,^^13^^,^^25^^,^^26^ Vaccination as part of targeted interventions increased oral cholera vaccination coverage in our setting, especially in the slums of Mokolo, in Afan Mabe and at the central prison of Kribi. Here, surplus doses of oral cholera vaccine obtained from the global stockpile for a mass vaccination campaign, were made available by the health ministry. Due to vaccine shortages, oral cholera vaccination was possible in only five out of eight targeted interventions, with poor coverage in the Petit Paris area. Unfortunately, the global oral cholera vaccine stockpile only has mechanisms to obtain oral cholera vaccine for preventive and reactive mass vaccination campaigns but not for case-area targeted interventions.^27^ *Médecins Sans Frontières* and other organizations thus rely on obtaining their own supply of oral cholera vaccine for targeted interventions.^28^

In the context of the COVID-19 pandemic, there has been growing concerns about vaccine safety among the public.^29^ Although we did not collect data on vaccine hesitancy in a formal way, anecdotally some people were reluctant to receive oral vaccine or antibiotic prophylaxis. The reasons for refusal were most often associated with the fear that this would be a strategy to escalate COVID-19 transmission. However, these people were accepting of the distribution of soap bars and chlorine tablets. Given the limited stocks, the distribution of water storage cans was based on household assessment.

Full packages of case-area targeted interventions include antibiotics used for prophylaxis of close household contacts.^10^^,^^30^ However, due to concerns about emergence of resistant *V. cholerae* strains,^31^^,^^32^ the Global Task Force on Cholera Control only recommends selective antibiotic chemoprophylaxis for closed populations at high risk of infection (such as prisons) and not community-wide chemoprophylaxis.^22^ For these reasons, in our settings, chemoprophylaxis was done in the central prison and reserved for close contacts in the primary households who had not previously received a dose of oral cholera vaccine. In addition to protecting unvaccinated close contacts, antibiotics may also have an important role in quickly protecting individuals from infection since they act faster than oral cholera vaccine.^15^

Implementation of targeted interventions in Kribi was supported by *Médecins Sans Frontières*, who covered the expenses related to human resources and physical resources (excluding chlorine tablets). The total cost of the intervention was 23 000 United States dollars. However, this amount was not the actual cost as some items were provided by *Médecins Sans Frontières* including drugs, rapid diagnostic tests, boots, vehicles, fuel and tablets. With good coordination among stakeholders, targeted interventions can be easily replicated in low-resource settings especially at the beginning or at the end of an outbreak, thereby minimizing the cost. In Cameroon, the health ministry has led individual targeted interventions in three other regions during the ongoing outbreak in 2022, with the support of partners. The main challenges to scaling-up interventions in each region include ensuring widespread availability of rapid diagnostic tests, vaccines and funds; achieving timely deployment of interventions; and instituting decentralized and sustainable systems.

Our experience shows that, despite some challenges, a full package of targeted interventions with oral cholera vaccination could be promptly implemented at the tail-end of a cholera outbreak. Despite limited evidence, targeted interventions seem to be a promising alternative strategy in the cholera control toolkit. For smooth implementation of targeted interventions during subsequent cholera epidemics worldwide, we recommend scaling-up the availability of single-dose oral cholera vaccination, antibiotics and water, sanitation and hygiene items. Our study should stimulate deeper investigations of the effectiveness of case-area targeted interventions throughout the cycle of cholera outbreaks.
